# Intake of Products Containing Anthocyanins, Flavanols, and Flavanones, and Cognitive Function: A Narrative Review

**DOI:** 10.3389/fnagi.2021.640381

**Published:** 2021-09-03

**Authors:** Samantha L. Gardener, Stephanie R. Rainey-Smith, Michael Weinborn, Catherine P. Bondonno, Ralph N. Martins

**Affiliations:** ^1^School of Medical and Health Sciences, Edith Cowan University, Joondalup, WA, Australia; ^2^Australian Alzheimer’s Research Foundation, Ralph and Patricia Sarich Neuroscience Research Institute, Nedlands, WA, Australia; ^3^Centre for Healthy Ageing, Health Futures Institute, Murdoch University, Murdoch, WA, Australia; ^4^School of Psychological Science, The University of Western Australia, Crawley, WA, Australia; ^5^Medical School, Royal Perth Hospital, The University of Western Australia, Perth, WA, Australia; ^6^Department of Biomedical Sciences, Faculty of Medicine and Health Sciences, Macquarie University, Sydney, NSW, Australia

**Keywords:** flavonoids, cognition, Alzheimer’s disease, anthocyanins, flavanols, flavanones, dementia

## Abstract

The purpose of this review is to examine human research studies published within the past 6 years which evaluate the role of anthocyanin, flavanol, and flavanone consumption in cognitive function, and to discuss potential mechanisms of action underlying any observed benefits. Evidence to date suggests the consumption of flavonoid-rich foods, such as berries and cocoa, may have the potential to limit, or even reverse, age-related declines in cognition. Over the last 6 years, the flavonoid subgroups of anthocyanins, flavanols, and flavanones have been shown to be beneficial in terms of conferring neuroprotection. The mechanisms by which flavonoids positively modulate cognitive function are yet to be fully elucidated. Postulated mechanisms include both direct actions such as receptor activation, neurotrophin release and intracellular signaling pathway modulation, and indirect actions such as enhancement of cerebral blood flow. Further intervention studies conducted in diverse populations with sufficient sample sizes and long durations are required to examine the effect of consumption of flavonoid groups on clinically relevant cognitive outcomes. As populations continue to focus on adopting healthy aging strategies, dietary interventions with flavonoids remains a promising avenue for future research. However, many questions are still to be answered, including identifying appropriate dosage, timeframes for intake, as well as the best form of flavonoids, before definitive conclusions can be drawn about the extent to which their consumption can protect the aging brain.

## Introduction

Flavonoids are a class of polyphenols found in plant-based foods and are categorized into six major subclasses: anthocyanins, flavanols, flavonols, flavanones, flavones, and isoflavones (Spencer, 2008). Fruits and vegetables, wine, tea, and cocoa contain high concentrations of flavonoids (Somerset and Johannot, 2008). Structurally, flavonoids consist of two aromatic carbon rings (benzopyran A and C rings), and a benzene (B) ring, and may be divided into various subgroups based on the degree of the oxidation of the C ring, the hydroxylation pattern of the ring structure, and the substitution of the three-position. Once ingested, flavonoids undergo extensive metabolism in the small and large intestine, in the liver, and within cells, resulting in forms in the body distinct from those consumed in foods (Manach et al., 2005).

The reduction in risk of cardiovascular disease and some cancers afforded by intake of flavonoids is well known (Kampa et al., 2007; Chong et al., 2010), and more recently, there has been attention directed toward their potential to improve cognitive performance in older adults, and thereby potentially protect against neurodegenerative diseases such as Alzheimer’s disease (AD) (Bergland et al., 2019; Chai et al., 2019). In light of the projections indicating rapid increases in the prevalence of AD, and in the absence of successful treatments, alternative measures to slow the development and progression of the disease are imperative: flavonoid intake is being investigated as one such alternative measure.

Studies using animal models [reviewed in Nehlig (2013), de Andrade Teles et al. (2018), Hajialyani et al. (2019)] have demonstrated that flavonoids may slow or even reverse the effects of aging and dementia of which AD is the most common form. For example, in a rat model of aging which demonstrates inflammation, oxidative stress, memory and synaptic dysfunction, intraperitoneal injection of 100 mg/kg anthocyanins daily for 7 weeks, significantly improved spatial learning and memory ability, as well as short term memory (Rehman et al., 2017). Moreover, spatial learning and memory impairments were prevented in 4-month-old male senescence-accelerated mouse prone-8 (SAMP8) animals, orally administered 0.05 or 0.1% green tea catechins in drinking water for 6 months (Li et al., 2009). SAMP8 is typically characterized by early onset of learning and memory deficits along with spontaneous overproduction of soluble Aβ in the brain, a hallmark of AD.

While existing animal studies provide important insights into the neuroprotective properties of flavonoids and their underlying mechanisms, these studies cannot provide information regarding potential effects on more complex human cognitive functions such as abilities underpinned by language, and those supported by multifaceted organization of information, i.e., executive function. Consequently, clinical studies are required to determine if the beneficial effects observed in animal models are replicable in human populations.

The purpose of this narrative review is to examine human research studies published within the past 6 years (2014–2020 inclusive), to evaluate associations between intake of products containing the flavonoid subclasses of anthocyanins, flavanols, and flavanones and cognitive function (summarized in Supplementary Table 1 and Table 1). Potential mechanisms underlying any observed benefits will also be discussed. Studies preceding this timeframe have been comprehensively reviewed elsewhere; e.g., Spencer, 2010; Lamport et al., 2012; Crichton et al., 2013. We focused on the three subclasses of anthocyanins, flavanols, and flavanones as the effects of the additional three flavonoid subclasses (flavones, flavonols, and isoflavones) have not been reported in human studies published within the 6-year timeframe of this narrative review.

**TABLE 1:** Observational human studies published over the past 6 years investigating flavonoid consumption and cognitive outcomes.

**Title, reference**	**Cohort information**	**Study design**	**Flavonoids investigated**	**Outcome measure(s)**	**Main finding(s)**
Long-term dietary flavonoid intake and change in cognitive function in the Framingham Offspring Cohort (Shishtar et al., 2020)	*N* = 1779 Age: ≥45 Cognitively normal Framingham, MA, United States	Prospective cohort study Median 11.8-year follow-up	Total flavonoids, flavanones, flavan-3-ols, flavonoid polymers, flavonols, flavones, and anthocyanins	Verbal memory, visual memory, verbal learning, attention and concentration, abstract reasoning, language, visuoperceptual organization, and global function	Nominally significant trends observed between slower decline in global function, verbal and visual memory with higher flavonol and flavan-3-ol intakes; slower decline in visual memory with higher total flavonoid and flavonoid polymer intake; and slower decline in verbal learning with higher flavanol intake
The efficacy of cocoa polyphenols in the treatment of mild cognitive impairment: a retrospective study (Calabro et al., 2019)	*N* = 55 Age: 56–75 Amnestic MCI Messina, Italy	Retrospective cohort study over 1 year	Mexenion^®^ composed of cocoa polyphenols (240 mg/sachet)	MMSE	Participants were divided into two groups depending on change in MMSE score at 1-year follow-up: Group 1 = stable or improved, Group 2 = decreased by at least three points. Group 1 had a significantly higher rate of Mexenion^®^ intake than Group 2
Chocolate intake is associated with better cognitive function: The Main Syracuse Longitudinal Study (Crichton et al., 2016)	*N* = 968 Age: 23–98 Syracuse, NY, United States	Cross-sectional study	Chocolate intake divided into categories: less than once a week, once per week, more than once per week	Maine-Syracuse Longitudinal Study neuropsychological test battery: Composite domains global, visual-spatial memory and organization, scanning and tracking, verbal episodic memory, and working memory. Wechsler Adult Intelligence Scale Similarities Test MMSE	Chocolate consumption was significantly and positively associated with the global composite, visual-spatial memory and organization, scanning and tracking, verbal episodic memory, working memory, Wechsler Adult Intelligence Scale Similarities Test, and MMSE

*MCI, Mild Cognitive Impairment; MMSE, Mini-Mental State Examination.*

## Methods

A computer-based search of PubMed was conducted for relevant articles published between 2014 and 2020 inclusive, with cognitive function as the primary or secondary outcome. Search terms were: (flavonoids or anthocyanins or flavanols or flavanones) and (cognition or cognitive function). The search was limited to articles in humans, published in English. Studies that specifically focused on the cognitive effects of flavonoids either in extract or whole food form were included.

Titles and abstracts were screened by the first author to ensure they examined the relationship between one of the flavonoid subclasses of interest (anthocyanins, flavanols, or flavanones) and cognition in adults. Articles were included if they reported prospective, retrospective or intervention studies and were either cross-sectional or longitudinal in nature. There were no strict criteria relating to the inclusion of controls or the quality of these controls with respect to matching for other macro or micronutrients. Studies were excluded if they; (1) were not peer-reviewed, (2) were a conference proceeding abstract, (3) were a review or theoretical article, (4) were published in a language other than English, (5) were conducted in animal models, or (6) were conducted in children (age <18 years). The reference lists of included articles were screened and additional articles which met inclusion criteria were added via hand search. Included studies are presented in Supplementary Table 1 and Table 1.

## Anthocyanins

Anthocyanins are a class of flavonoids concentrated in purple and red fruits. In summary, we have reviewed four acute studies (<24 h) and eight chronic studies (duration between 12 and 24 weeks) investigating cherry, blackcurrant, bilberry, and blueberry anthocyanins in participants with varying cognitive status, ranging from cognitively normal (both young and older adults), self-perceived cognitive decline, and objectively determined mild cognitive impairment (MCI), to diagnosed dementia.

A 12-week randomized clinical trial conducted in 49 adults (24 intervention group, 25 control group) over the age of 70 years with mild to moderate AD, found consumption of 200 ml/day of cherry juice resulted in improvements in verbal fluency, short term memory, and long term memory compared to the control group, which consumed a juice with negligible anthocyanin content. The intervention group also had a reduction in systolic blood pressure, with a trend for reduction in diastolic blood pressure. However, markers of inflammation (CRP, C-reactive protein; IL-6, Interleukin 6) were not altered (Kent et al., 2017); suggesting either the mechanism of action of anthocyanins is not through the inflammatory pathway, or perhaps more likely, that the advanced disease stage which is accompanied by significant pathological hallmarks and inflammation, masked any potential anti-inflammatory effects conferred by the cherry juice. Of note, pre-clinical studies have indicated that anthocyanins from berry fruits may require only a few weeks to accumulate in brain regions associated with cognition (Willis et al., 2009). Twelve-week consumption of two cups of Montmorency tart cherry juice per day resulted in better performance on the paired associates learning task which assesses visual memory (mean difference of −8.5; 95% −12.5 to −4.5; *p* = 0.02), and significantly better movement time scores on the Reaction Time test which measures speed of response (mean difference of −10.4; 95% −13.4 to −7.5; *p* = 0.03), compared to the control group. This study was conducted in 37 adults aged between 65 and 80 with normal cognitive function. There were no significant improvements in working memory and strategy use, and visual attention. It is, however, important to note, that the intervention group had significantly decreased physical activity level whilst the control group had significantly increased cholesterol intake over the intervention period (Chai et al., 2019): factors which may have impacted the outcome measures.

In contrast, Caldwell et al. (2016) found no change in acute memory following consumption of a 300 ml serving of cherry juice, either administered as a single serving at time “0,” or three 100 ml servings at “0,” 1, and 2 h, with cognitive testing occurring at time “0” and 6 h post-intervention, in six young healthy participants, five older adults, and five older adults with dementia. The cognitive tests assessed verbal learning and memory, higher executive function, and speed of processing. The serving of cherry juice contained approximately 55 mg of anthocyanins; a lower dose compared to other food-based studies. The cognitive tests administered may not have adequately assessed the cognitive domains affected by acute anthocyanin consumption. Moreover, testing at 2 rather than 6 h post-intervention may better reflect the physiological absorption of anthocyanins in the small intestine.

In an intervention trial undertaken in 36 healthy young participants (aged 18–35 years), individuals consumed one of three different treatment drinks once, with at least 7 days of washout between consumption of each drink. The drinks contained either 0 mg of polyphenols (control), 525 ± 5 mg of polyphenols per 60 kg of bodyweight from an anthocyanin-enriched blackcurrant extract, or from 142 ml of a cold-pressed blackcurrant fruit juice. Participants underwent 10 min of cognitive testing before consumption of each treatment drink, followed by additional cognitive testing 60 min post-consumption, comprising seven repetitions of the 10-min battery. Improvements in accuracy on the Rapid Visual Information Processing task were found after supplementation with the extract drink (*p* = 0.011), and improvements in reaction time on the digit vigilance task after supplementation with the juice treatment, at repetitions 1 (*p* = 0.028), 4 (*p* = 0.011), and 7 (*p* = 0.038). The juice drink also induced a number of neuroendocrinological and physiological effects including increase in blood glucose concentration compared with control. These results suggest that the degree of processing and the cultivar of blackcurrant fruit used substantially alters the neuroendocrinological and cognitive benefits conferred (Watson et al., 2015).

A pilot study measuring cognition as a secondary endpoint following 16-week consumption of two Medox^®^ capsules per day (total daily intake of 320 mg anthocyanin from bilberry and blackcurrants) found improved verbal memory scores in the subdomains of learning (*p* = 0.016), recall (*p* < 0.001), and recognition (*p* = 0.047). Improved cognitive speed (*p* < 0.001) and inhibition (*p* = 0.044) were also observed, whilst no improvements in executive functioning were seen. The intervention group was comprised of eight participants with MCI, and 19 with stable non-obstructive coronary artery disease, as the study was designed to investigate supplementation in those with increased risk of dementia. However, the control group did not undergo cognitive testing; consequently, these results should be interpreted with caution due to the lack of a comparison group (Bergland et al., 2019).

Several randomized, double-blinded, placebo-controlled studies to evaluate the effects of blueberry consumption on cognition have been conducted. In 112 adults aged 65–80, Whyte et al. (2018) investigated the effect of 6-month consumption of; (1) whole wild blueberry powder containing 1.35 mg total anthocyanin per dose (WBP500), (2) whole wild blueberry powder containing 2.7 mg total anthocyanin per dose (WBP1000), or (3) purified extract containing 7 mg total anthocyanin per dose (WBE111), compared to placebo. Cognitive testing occurred at baseline, 3 and 6 months using a battery targeting episodic memory, working memory, and executive function. At the 3-month testing point, delayed word recognition and short-term spatial episodic memory were significantly better in the WBE111 treatment group compared to control. There were no significant differences at 6 months, or with consumption of the WBP500 and WBP1000 at any timepoint. It should be noted, however, that the quantity of anthocyanins administered is relatively small compared to other studies. Miller et al. (2018) recruited 13 men and 24 women between the ages of 60 and 75 to consume either 24 g of freeze-dried blueberries (containing 19.2 mg/g anthocyanins), or placebo for 90 days, with cognitive testing conducted at baseline, 45, and 90 days. Participants in the blueberry group showed a greater reduction in switch stimuli errors (measure of executive function) across visits compared to the control group [*F*(2,70) = 3.587; *p* = 0.033; η*p*^2^ = 0.09]. Participants in the blueberry group also made fewer repetition errors on the California Verbal Learning Test free recall (measure of verbal memory) at 90 days compared to baseline, whilst the control group demonstrated increased errors compared to baseline [*F*(1,35) = 5.024; *p* = 0.031; η*p*^2^ = 0.126]. No effect of the intervention was seen on tests of psychomotor speed, short-term memory, spatial cognition, or attention.

A single-blind, randomized, placebo-controlled, between-subjects study investigated executive function changes over a 6-h period following consumption of a 400 mL smoothie containing equal blueberry, strawberry, raspberry, and blackberry (*n* = 20), or placebo (*n* = 20) in 20–30 years old. Following berry intervention, participants maintained accuracy in executive function, up to and including the 6-h testing point, measured using the Modified Attention Network Task (MANT) and Task Switching Task (TST). Participants demonstrated quicker response times in the MANT conducted at the 2- and 4-h post-smoothie consumption cognitive assessments, and in the TST at 6-h post-smoothie consumption. The results of this study suggest that berry-related benefits were more evident during periods of fatigue, with placebo participants showing decreased performance across the 6 h as they became cognitively fatigued. Whilst the placebo was matched to the intervention for sugar content and sweetness, it was not matched for berry flavor, thus participants may have guessed which treatment they received, potentially leading to performance being influenced by placebo effect (Whyte et al., 2019).

A cross-over randomized controlled trial was conducted in 18 older adults (aged 60–75) using a flavonoid-rich blueberry beverage drink (579 mg of antho- and pro-cyanidins), or sugar-matched control. Cognitive function was assessed at baseline, 2- and 5-h post-beverage consumption. Whilst there was no significant effect of consumption of the flavonoid-rich beverage on global cognition compared to the control drink, there was a decline in performance in the control group from the 2-h to the 5-h assessment [*F*(1,36) = 4.60; *p* = 0.04], which was not observed in the intervention group. There were also no significant differences between groups in performance on the 14 tasks that comprised the global cognitive function score; although some associations were approaching significance, suggesting that the study may be underpowered (Dodd et al., 2018).

Sixty-five men and women aged 62–80 years completed a 24-week randomized double-blind, placebo-controlled trial consuming either daily fish oil, daily blueberry (providing 269 mg anthocyanin per day), both fish oil and blueberry, or control. Participants all reported mild, age-related cognitive decline, but lacked a diagnosis of MCI or dementia. No effect on motor speed, working memory, learning and retention, or lexical access was observed for the fish oil group, compared to placebo, at the end of the 24-week intervention, or at 48-week follow-up. There was improved discrimination in recognition memory for the blueberry treated group at 24-weeks, indicating improved resistance to interference of extraneous material in memory; however, this benefit was not maintained at week 48. Unexpectedly, there was no effect on any cognitive domain of the combined fish oil and blueberry treatment (McNamara et al., 2018).

In a randomized, double-blind placebo-controlled trial of 16 older adults (aged 68–92 years) with MCI, functional magnetic resonance imaging (MRI), conducted during a working memory task, was assessed pre- and post- 16-weeks of blueberry supplementation (providing 269 mg anthocyanins daily). Whilst there was no clear indication of working memory enhancement associated with supplementation, enhanced neural response during the working memory task was observed in the blueberry treatment group compared to placebo. Accuracy for the treatment group in the *1*-back task was trending toward significance (*p* = 0.08; participants had to recognize when the currently displayed letter was the same as the letter displayed one item previously), with the authors stating that the large observed effect size (*d* = 1.02) indicated that increasing the sample size from 8 to 17 per group would have been sufficient to achieve significance at *p* = 0.05 with power = 0.80 (Boespflug et al., 2018).

Change in performance on cognitive tests was not significantly different between placebo and blueberry supplementation treatment groups in a 12-week double-blind randomized controlled trial of 26 cognitively normal participants aged over 65 years. Participants underwent computerized tests of psychomotor function, visual processing, executive function, verbal and spatial memory, and working memory. The percentage change in performance on the *2*-back test did, however, show weak evidence for improvement in the blueberry treatment group compared to placebo (Bowtell et al., 2017).

Of the 12 studies reviewed in this section, six reported improvements in at least one cognitive domain following anthocyanin consumption (four chronic studies and two acute), whilst six studies showed no improvement or change compared with placebo (four chronic studies and two acute). Cognitive domains impacted by anthocyanin intake included verbal fluency, short term memory, long term memory, visual memory, speed of response, accuracy, verbal memory, and executive function. Several cognitive domains including verbal memory and executive function were observed as being both impacted by anthocyanin intake in some studies but not affected in others; the possible reasons for these disparate results including cohort differences and dose are discussed in the “Discussion.”

## Flavanols

Chocolate and cocoa products are a rich source of flavonoids with flavanols, and in particular epicatechin, being the most common type present in cocoa. High levels of flavanols are also found in tea, red wine, and fruits such as grapes and apples. In summary, we have reviewed two observational studies, six acute studies (<24 h) and seven chronic studies (duration between 1 and 6 months) investigating flavanols from fruit and vegetables, grape juice, cocoa, and apples in mainly cognitively normal participants (both young and older adults), with one MCI cohort.

A recently published intervention study of 211 healthy older adults (50–75 years) found a significant intake level-dependent treatment effect, compared to placebo, on hippocampal-dependent list-learning performance, following 12-weeks of flavanol intake (260, 510, or 770 mg/day cocoa flavanols). However, object recognition and prefrontal cortex-dependent list sorting performance did not improve. Eight weeks post-cessation of flavanol consumption, there were no observable effects remaining on list-learning performance in the intervention group compared to placebo. Notably, the object recognition task was newly developed, and data analysis suggested the task was so difficult that the majority of participants performed no better than chance; thus, failure to observe associations with flavanol intake may have been due to the psychometric properties of this outcome measure (Sloan et al., 2021).

Gratton et al. (2020) showed that flavanol intake can improve efficiency in blood oxygenation during hypercapnia (carbon dioxide-dependent increase in cerebral blood flow), and that this is likely to contribute to improvements in cognitive function, only when cognitive demand is high; significant associations were only observed in the Double Stroop Task and not in the standard Stroop Task. This study was conducted in 18 healthy males (18–45 years) using an acute, randomized, placebo-controlled, double-blind, crossover design, with high or low cocoa flavanol drink, and cognitive function assessed pre- and 2-h post-consumption (using a Modified Stroop Task). One advantage of this study is the intervention and placebo were matched for macronutrient and micronutrient content, including caffeine. With the cohort comprising only young males, future work should be extended to include females as well as older at-risk populations.

A study retrospectively analyzing data from 55 participants with amnestic MCI (29 males and 26 females, aged 56–75) found dietary supplementation with cocoa flavonoids was associated with slowed cognitive decline. Specifically, participants were divided into two groups depending on change in Mini-Mental State Examination (MMSE) score at 1-year follow-up (Group 1 = stable or improved, Group 2 = decreased by at least three points). Intake of the commonly used nutraceutical Mexenion^®^ was examined. Mexenion^®^ is composed of cocoa polyphenols (240 mg/sachet), *Bacopa monnieri* (110 mg/sachet), group B vitamins, vitamin E, and folic acid. Group 1 had a significantly higher rate of Mexenion^®^ intake compared to Group 2 which demonstrated decline in cognitive function at 1-year follow-up [χ2 = 13.79, *p* < 0.001; 29]. However, given the formulation of Mexenion^®^, it is not possible to determine directly if the cocoa polyphenols present were responsible for the observed benefit.

Neshatdoust et al. (2016) conducted a randomized, controlled, double-masked, crossover dietary intervention involving 40 cognitively normal participants, aged 68.3 ± 3.0, who consumed either a high flavanol cocoa drink (494 mg flavanols) or a low flavanol cocoa drink (23 mg flavanols) for 28 days. After the first intervention period, there was a 4 week wash out prior to switching treatment. A cognitive battery measuring global executive function, and blood sample collection was completed pre-intervention, post-first intervention, and at the end of the study. Intake of the high flavanol cocoa drink resulted in significantly better performance on the global executive function measures (*p* < 0.01), and an increase in serum brain-derived neurotrophic factor (BDNF) levels (*p* < 0.01), relative to the low flavanol cocoa drink intervention. BDNF is a member of the neurotrophin family of growth factors that help to stimulate and control neurogenesis. The authors additionally conducted a randomized, controlled, dose-dependent, parallel designed trial in 154 participants (aged 26–70 years) with a high flavonoid fruit and vegetables (F&V) diet, or a low flavonoid F&V diet, compared to a habitual control diet. Participants consumed two portions of F&V for the first 6 weeks, followed by four portions for the next 6 weeks, and finally six portions for the remaining 6 weeks, this equated to 3, 6, and 7 mg/d in the low flavonoid intake group, and 49, 121, and 198 mg/d in the high flavonoid intake group. All F&V were provided to the participants and effort was taken to match the intake of other bioactive components of F&V such as carotenoids, vitamin C, and folate. Cognitive testing and blood sample collection occurred at weeks 0, 6, 12, and 18. Results showed significantly better performance on the global executive function measures at 12 and 18 weeks for the high flavonoid F&V diet group relative to both the low flavonoid F&V diet and the habitual control diet (*p* < 0.01). Furthermore, serum BDNF was significantly higher at 18 weeks in the high flavonoid F&V diet group relative to the habitual control diet (*p* < 0.001). The authors concluded that increased brain and peripheral BDNF expression could be mediating the improvements observed in cognitive function (Neshatdoust et al., 2016).

Flavanol-rich chocolate has been shown to counteract the effects of sleep deprivation on working memory in women but not men. Sixteen women and 16 men participated in a randomized, double-blind, crossover study, undergoing four sets of cognitive testing, two after a night of undisturbed sleep and two after a night of total sleep deprivation, with a week between each of the four testing sessions. Participants consumed either a flavanol-poor chocolate bar or a flavanol-rich chocolate bar before each of the testing sessions. Accuracy in the *2*-back task was significantly higher in the sleep deprivation condition when the flavanol-rich chocolate was consumed, compared to the flavanol-poor chocolate, in women only (*p* = 0.04). Consumption of the flavanol-rich chocolate also produced positive effects on blood pressure, flow-mediated dilation, and pulse-wave velocity, which were all impaired following sleep deprivation (Grassi et al., 2016).

The dentate gyrus (DG), a brain region in the hippocampal formation, demonstrates functional decline with aging. In a controlled randomized trial of 37 cognitively normal adults aged 50–69 years, Brickman et al. (2014) showed that consumption of a high cocoa-containing diet for 3 months enhanced performance on a novel object recognition task which localizes function to the DG (high flavanol reaction time = 1,997 ms; low flavanol reaction time = 2,627 ms; t31 = 2.17; *p* = 0.038; Cohen’s *d* = 0.816). There was no effect of the diet on delayed retention. The high flavanol group consumed two 450 mg supplements per day, and the low flavanol group consumed a total of 45 mg divided into two doses. The high flavanol group also demonstrated a significant increase in cerebral blood volume in the DG and the downstream subiculum in the body of the hippocampus, with the changes in object recognition task performance correlating with these cerebral blood volume increases. The authors concluded that DG dysfunction is a driver of age-related cognitive decline, and flavanol consumption may be a method to ameliorate this process.

The effect of consumption of a high flavanol cocoa drink compared to a low flavanol cocoa drink on cognitive function was investigated in 90 cognitively normal elderly individuals in a double-blind, controlled, parallel-arm study. Participants were randomly assigned to groups consuming either a drink containing 993 mg (high flavanol), 520 mg (intermediate flavanol), or 48 mg (low flavanol) cocoa flavanols, daily for 8 weeks. No change in MMSE score in response to the three treatments was observed. However, better performance on the Trail Making Test A and B was observed after consumption of the high and intermediate flavanol drinks, compared to the low flavanol drink (*p* < 0.001). Verbal Fluency Test scores were also significantly better after consumption of the high flavanol drink, compared to the intermediate and low flavanol drinks (*p* < 0.001). Additionally, significant improvements in insulin resistance, blood pressure, and lipid peroxidation were seen in the high and intermediate flavanol groups, in comparison to the low flavanol group; highlighting potential mechanistic pathways through which cocoa flavanols exert positive effects on cognition (Mastroiacovo et al., 2015).

By contrast, Massee et al. (2015) found daily consumption of cocoa supplementation (250 mg catechin polyphenols and 5.56 mg caffeine), for 4 weeks, had no effect on cognitive performance in 40 young cognitively normal adults. However, acute consumption, with cognitive testing conducted 3 h after first supplementation, was associated with significant improvement on Serial Sevens performance, in the first of three cycles of cognitive testing. Notably, the intervention duration was half that of Mastroiacovo et al. (2015), whilst the quantity of flavanols consumed was considerably less than the intermediate flavanol drink in that study. Additionally, participants in the current study were considerably younger with mean age 24.13 ± 4.47, and it is possible that by starting cognitive testing 3 h post-flavanol consumption the peak in flavanol concentration may have been missed.

A study investigating the effect of cocoa flavanol intake combined with exercise on executive function found that, whilst flavanol intake increased cerebral oxygenation during the executive function task, there was no impact on cognitive performance. Furthermore, exercise alone improved cognitive function, however, there was no additive effect of the flavanol consumption when combining the two conditions. The executive function task comprised the Stroop test, and potentially this short cognitive task was not sufficiently challenging for the young, healthy cohort to show improvements. The cohort comprised 12 males with a mean age of 30 ± 3 years, and the study was randomized, double-blind, and crossover in design (flavanol/placebo intake occurred 100 min preceding a 30 min time trial), with flavanol consumption of 904 mg (treatment), or 15 mg (control drink) (Decroix et al., 2016). Furthermore, a study investigating cognitive function an hour after consumption of either a low flavanol containing white chocolate, a medium flavanol containing milk chocolate, or a dark chocolate containing high flavanol levels, found no improvements on a computerized test battery (Cogstate Ltd.). The authors did, however, observe enhanced vascular endothelial function following the high flavanol chocolate condition which was reflected by improvements in brachial artery flow-mediated dilation. The cohort was limited to 12 post-menopausal women, but included strict control of the type, composition, and energy content of the chocolate used to ensure consistency between conditions (Marsh et al., 2017).

A cross-sectional study undertaken on 968 community-dwelling participants between the ages of 23 and 98 years, drawn from the Maine-Syracuse Longitudinal Study, showed that eating chocolate more than once a week was associated with better performance on a global cognitive composite (*p* < 0.001), as well as better visual-spatial memory and organization (*p* < 0.001), working memory (*p* = 0.048), scanning tracking (*p* < 0.001), and abstract reasoning (*p* < 0.001), compared to eating chocolate less than once a week. There were no associations observed between chocolate intake and verbal memory. An important point to note, however, is that chocolate intake was not differentiated according to type, i.e., dark, milk, or white, all of which contain varying quantities of flavanols (Crichton et al., 2016).

Apple skin contains high concentrations of the flavanol (−)-epicatechin and the flavonol quercetin. In a randomized, controlled, crossover design study of 30 cognitively normal participants, with mean age 47.3 ± 13.6, individuals received the following treatments; (1) control: low flavonoid, low nitrate, (2) apple: high flavonoid, low nitrate, (3) spinach: low flavonoid, high nitrate, and (4) apple + spinach: high flavonoid, high nitrate (the authors were also assessing effects from nitrates). There was a minimum washout period of 1 week between treatments. Relative to control, all treatments augmented nitric oxide status acutely, but had no effect on cognitive function 150 min post-intervention. The cognitive battery assessed “Quality Working Memory,” “Power of Attention,” and “Continuity of Attention,” and cognitive effects may have been evident with tasks measuring additional domains. The “younger” age range of participants and absence of cognitive impairment may also account for the lack of observed effect. Furthermore, this study assessed the acute effects of flavonoid intake, raising the possibility that longer-term consumption is required to induce cognitive benefit (Bondonno et al., 2014).

A 6-month bicentric, randomized, double-blind, placebo-controlled trial conducted in 215 cognitively normal participants (aged 60–70) receiving 600 mg/day of a flavanol-rich extract from grape and blueberry, demonstrated improvements in verbal episodic and recognition memory (VRM)-free recall in the treatment group, with no effect observed on Cambridge Neuropsychological Test Automated Battery (CANTAB) Paired Associate Learning (PAL), a measure of visual-spatial learning and episodic memory. However, there was a subgroup with “advanced cognitive decline” who showed better VRM-delayed recognition, post-flavanol treatment, only when the cohort was stratified into quartiles using baseline PAL score. The authors summarized that the study demonstrated a positive effect of flavanol intake on episodic memory in otherwise cognitively normal older adults with a lower level of memory performance (Bensalem et al., 2019).

Moreover, a randomized, placebo-controlled, double-blind, counterbalanced-crossover study of 20 healthy young adults (mean age 21.05 ± SD 0.89) found 230 mL of purple grape juice significantly improved reaction time, compared to placebo, on a composite attention measure (*p* = 0.047), 20 min after consumption. However, there was no effect on attention accuracy, or on measures of memory (Haskell-Ramsay et al., 2017).

Of the 15 studies reviewed in this section, 11 found improvements in at least one cognitive domain following flavanol consumption (two observational studies, six chronic, and three acute intervention studies). Of these 11 studies, flavanol intake was associated with improvements in global cognition as well as the cognitive domains of visual-spatial memory and organization, working memory, abstract reasoning, accuracy, reaction time, executive function, episodic memory, verbal fluency, and recognition memory. There were no common methodological approaches amongst the studies reporting positive effects compared to those reporting no effect, with a wide range of age groups included, varying flavanol doses utilized and two studies incorporating the additional variables of sleep deprivation and exercise. Factors likely impacting the varying results reported are discussed in greater detail in the “Discussion.”

## Flavanones

Orange juice is one of the most commonly consumed juices throughout the world and is a rich source of flavanones, particularly hesperidin and narirutin. As one of the most readily absorbed flavonoid subclasses, flavanones have been shown to cross the blood-brain barrier. In summary, we have reviewed two acute studies (<24 h) and one chronic study (duration 8 weeks) investigating flavanones from orange juice in cognitively normal participants (both young and older participants), and one prospective cohort study examining long-term intake of total and the six classes of dietary flavonoids over a follow-up period of up to 15 years, in dementia-free individuals aged 45 years and over.

High flavanone (HF; 305 mg) 100% orange juice and a low flavanone (LF; 37 mg) orange-flavored cordial were consumed daily for 8 weeks by 37 cognitively normal older adults (mean age 67 years) in a crossover, double-blind, randomized study, with a 4 weeks wash out period between treatments. After 8 weeks of consuming the HF drink, global cognitive performance was significantly better than after the LF drink intervention (*t* = 2.86; *p* < 0.05). In other cognitive areas, the HF drink reduced the decline in performance associated with LF beverage consumption. Further, performance was better when the HF drink was consumed in the first arm of the trial than when the LF drink was consumed first, indicating effects of the HF drink may have continued into the second arm of the intervention (Kean et al., 2015).

The effect of drinking a HF orange juice (272 mg) compared with placebo has been investigated acutely, with cognitive testing conducted at 2- and 6-h post-consumption, in a randomized, double-blind, counterbalance study, with a 2-week wash out period. This study was conducted in 22 healthy males between 30 and 65 years of age. The HF drink contained 220.46 mg hesperidin, 34.54 mg narirutin, and other flavonoids (17.14 mg). Performance on the Simple Finger Tapping test, a measure of psychomotor speed (involves pressing the “2” key as many times with the index finger over a 10 s period) was better, following the HF drink, relative to placebo [*F*(1,20) = 8.32; *p* < 0.01]. Additionally, the change in performance from baseline to 6 h post-consumption on Continuous Performance Task accuracy, a measure of psychomotor speed, was significantly different across the treatments, with fewer errors observed when the HF drink had been consumed. This test involves random presentation of letters at a rate of one every 250 ms for a duration of 6 min, and participants are required to press the space bar when a letter other than “X” is presented (Alharbi et al., 2016).

Lamport et al. (2016) investigated acute effects of HF juice consumption (orange and grapefruit juice) compared to a concentrated cordial drink containing zero flavonoids. The HF drink contained considerably less total flavanones than the previously mentioned studies, with 70.5 mg present (42.15 mg hesperidin, 17.25 mg naringin, 6.75 mg narirutin, and 4.3 mg caffeic acid); however, the drink also contained additional flavanones in greater proportions, for example naringin. Twenty-four young adults between 18 and 30 years of age underwent cognitive testing at baseline and 2 h post-drink consumption, and a further 16 participants underwent functional MRI assessment at baseline, 2- and 5-h post-drink consumption to measure cerebral blood flow (CBF). The authors observed significantly improved performance on the Digit Symbol Substitution Test (DSST) 2 h after consumption of the HF drink, with no improvements following consumption of the control drink [*F*(1,23) = 10.76, *p* < 0.01]; however, other cognitive tests showed no improvements. Regional perfusion in the inferior frontal gyrus and middle frontal gyrus of the right hemisphere was significantly higher 2 h post-consumption of the HF drink compared with the control drink. There were no differences in regional perfusion 5 h after consumption of either drink. The inferior frontal gyrus has been implicated in tasks that require inhibition, planning, decision making, and other aspects of executive function, examined by the DSST. Stronger cognitive effects could have occurred more than 2 h post-drink consumption, as was seen in the previously mentioned studies, yet this was not assessed in the current study. However, increased CBF was observed at 2 h and not 5 h post-consumption in the current study, suggesting that the time course by which flavonoids in orange and grapefruit juice exert their effect may differ from orange juice alone. It is not possible, however, to determine a direct link between increased CBF and better cognitive function in the current study due to the separate cohorts utilized to investigate these outcomes.

All three studies reviewed thus far in this section reported improvements in at least one cognitive domain following flavanone consumption (one chronic study and two acute). Global cognition as well as the cognitive domains of psychomotor speed, and executive function were positively impacted by flavanone consumption. All three studies also reported no associations of flavanone intake with episodic memory and verbal memory. These three studies were conducted in cohorts of distinct age groups of cognitively normal adults, ranging from 30 to over 65 years. It appears therefore, that flavanone intake yields positive effects on cognition regardless of age. Further discussion of these results in conjunction with the results of the anthocyanin and flavanol studies is included in the “Discussion.”

Finally, a recent study assessed the association of long-term intake of total and six classes of dietary flavonoids (flavanones, flavan-3-ols, flavonoid polymers, flavonols, flavones, and anthocyanins) and decline in cognitive function, over a median follow-up period of up to 11.8 years, in 1779 participants who were dementia free, aged ≥45 years at commencement, and who attended at least two study assessments (approximately 4 years apart). The findings did not support a clear association, with nominally significant trends observed between (1) higher flavonol and flavan-3-ol intakes, and slower decline in global function, verbal and visual memory; (2) higher total flavonoid and flavonoid polymer intakes and slower decline in visual memory; and (3) slower decline in verbal learning with higher flavanol intake (Shishtar et al., 2020). The age range of participants, and sensitivity of cognitive measures used, may have impacted the study findings.

## Mechanisms of Action

Having a clear understanding of how these flavonoid subclasses are modulating cognitive function is required for furthering the use of these compounds as interventions which can be recommended by health professionals. For example, knowledge of a compound’s mechanism of action enables better dosing through monitoring of the compound’s effects on the target pathway in the patient. There is a relative paucity of data in humans, with the majority of mechanistic studies to date, having been undertaken in animal models. Nevertheless, these animal studies provide clues regarding potential mechanistic pathways in humans. Collectively, the published studies implicate a number of candidate mechanisms underlying the beneficial effects of consumption of these flavonoid subclasses on cognitive function, including, modulation of intracellular signaling pathways, altered CBF, and conferring protection against neurotoxins and neuroinflammation.

### Modulation of Signaling Cascades

The flavonoid subclasses of anthocyanins, flavanols, and flavanones modulate several neurological processes via their interaction with signaling pathways involved in neuronal survival and function, upregulation of proteins important for synaptic plasticity and neuronal repair, and inhibition of neuropathological processes which occur in brain regions typically implicated in AD pathogenesis.

**FIGURE 1:**
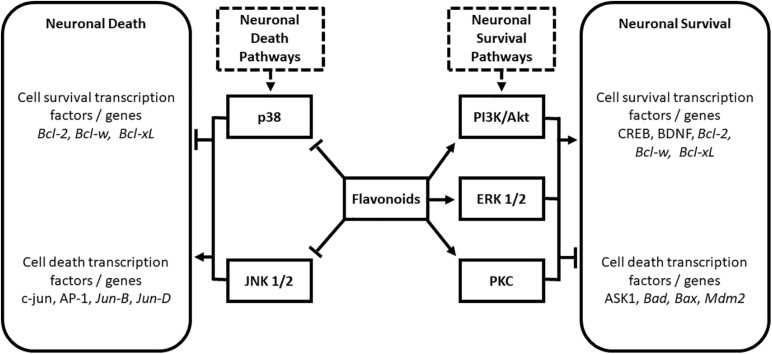
Modulation of neuronal death and survival pathways by the flavonoid subclasses of anthocyanins, flavanols, and flavanones. Modified from Solanki et al. (2015). AP-1, activated protein 1; ASK1, apoptosis signal-regulating kinase 1; Bad, BclxL/Bcl-2-associated death promoter; Bax, Bcl-2-associated X protein; Bcl-2, B-cell CLL/lymphoma 2; Bcl-w, BCL2-like 1; BDNF, Brain-derived Neurotropic Factor; CREB, cAMP response element-binding protein; ERK, extracellular signal-regulated protein kinase; JNK, c-Jun N-terminal kinase; Mdm2, murine double minute 2; PI3K/Akt, phosphatidylinositol-3 kinase/Protein kinase B; PKC, protein kinase C.

Various flavonoids interact with important cell survival signaling pathways including the phosphatidylinositol-3 kinase/Akt (PI3K/Akt), extracellular signal-regulated protein kinase (ERK), and protein kinase C pathways [PKC; (Levites et al., 2002; Spencer, 2007; Vauzour et al., 2007; Williams et al., 2008)]. Flavonoids have also been shown to modulate the cell death pathways mediated by p38 and c-Jun N-terminal kinase (Hwang and Yen, 2009) to confer protection against neurodegeneration. These interactions are summarized in Figure 1.

The activation of cell survival pathways by flavonoids involves upregulation of anti-apoptotic and pro-survival genes, and inhibition of pro-apoptotic proteins (Levites et al., 2002; Spencer, 2007). Targets include inhibition of apoptosis signal-regulating kinase 1 (ASK1; Vauzour et al., 2007), and non-transcriptional inhibition of BclxL/Bcl2-associated death promoter (Bad), BCL2-associated X protein (Bax), and murine double minute 2 (Mdm2; Levites et al., 2002). Flavonoids are also proposed to confer cell survival through activation of cAMP response element-binding protein (CREBP) phosphorylation (Williams et al., 2008) and increases in levels of BDNF (Williams et al., 2008). As mentioned previously, BDNF is a neurotrophin required for the development and maintenance of the nervous system (Schindowski et al., 2008). BDNF levels are known to decline during aging, and their levels have been shown to correlate with learning and memory (Garzon et al., 2002; Hwang et al., 2006; Laske et al., 2006). JNK and p38 are strongly linked to transcription-dependent apoptotic signaling (Mielke and Herdegen, 2000) via the activation of c-Jun and other activated protein 1 (AP-1) proteins including Jun-B and Jun-D (Behrens et al., 1999): flavonoids have been shown to impact these pathways at multiple levels to confer antiapoptotic activity (Ramiro-Puig et al., 2009).

The inflammatory cascade is believed to play a critical role in the development of chronic low grade inflammatory diseases such as AD. Reductions in blood levels of inflammatory markers such as CRP, IL-6, and tumor necrosis factor alpha (TNF-α) have been shown following flavonoid consumption, suggesting anti-inflammatory activity (Carey et al., 2014; Macready et al., 2014). Furthermore, flavonoids have the capacity to downregulate the activity of pro-inflammatory transcription factors such as NF-κB, nuclear factor erythroid 2-related factor 2 (Nrf2) and STAT (signal transducers and activators of transcription), through their influences on a number of glial and neuronal signaling pathways (Hamalainen et al., 2007; Risitano et al., 2014).

Mitochondria control cellular energy status, reactive oxygen species (ROS) production, and apoptosis, all of which are important for determining lifespan, and mitochondria are therefore, proposed to act as central organelles in the regulation of aging and neurodegeneration. Mitochondria constitute the major source of superoxide and other ROS within most tissues. AMP-activated protein kinase (AMPK), a key cellular regulator of energy metabolism, has been implicated in the regulation of mitochondria function. Damaged mitochondria are the major sources of ROS in cells and are implicated in many neurodegenerative diseases including AD. Activated AMPK can decrease intracellular ROS by inhibiting NADPH (nicotinamide adenine dinucleotide phosphate) oxidase activity, or by increasing the expression of antioxidant enzymes such as superoxide dismutase-2 and uncoupling protein-2 (Manczak et al., 2006). Flavonoids may ameliorate mitochondrial dysfunction and increase oxidative defense mechanisms by activating AMPK (Cordero-Herrera et al., 2013).

### Indirect Benefits to Cerebrovasculature

Consumption of flavonoids could improve cerebrovascular outcomes including CBF. Whilst the mechanisms require elucidation, they may be related to increases in the pool of bioavailable nitric oxide (NO; Bondonno et al., 2012), which is a key signaling molecule responsible for mediating vascular function changes (Heiss et al., 2007). Flavonoids may enhance NO levels by preventing NO breakdown, which could occur by a direct reaction with superoxide and other ROS and/or inhibition of the enzymes that produce these molecules [xanthine oxidase, lipoxygenase, and NADPH oxidase; (Nijveldt et al., 2001; Mladenka et al., 2010)]. One mechanism by which NO is synthesized is by the action of endothelial nitric oxide synthase (eNOS) on arginine, and flavonoids may enhance NO production by increasing eNOS activity or enhancing expression. Decreases in blood pressure, improvements in endothelial function (Bondonno et al., 2012), and improvements in CBF have all been observed in association with an increase in NO status (Francis et al., 2006). This is consistent with literature showing natural products that enhance brain metabolism or elevate CBF are effective at augmenting cognitive function during prolonged, effortful, cognitive processing (Owen and Sunram-Lea, 2011), with reduced blood flow to the brain associated with cognitive impairment (Farkas et al., 2002).

Moreover, there is evidence to show that increasing glucoregulatory control via improved insulin sensitivity can improve cognitive function, with intake of flavonoids being shown to increase insulin sensitivity, as well as insulin and glucose levels (Russo et al., 2019). Attenuation of decline in blood glucose concentrations has been observed following blackcurrant (Watson et al., 2015) and cranberry (Wilson et al., 2008; Torronen et al., 2010) consumption, which suggests glucose regulation may be an additional mechanism through which flavonoids confer cognitive benefit, particularly in the immediate postprandial period where the majority of improvements in cognitive performances were observed.

## Discussion

Evidence accumulated to date suggests the consumption of products containing anthocyanins, flavanols, and flavanones, such as berries and cocoa, throughout life, may have the potential to limit or even reverse age-related declines in cognition and memory, and potentially delay the onset and progression of neurodegenerative diseases such as AD. Over the last 6 years, the flavonoid subgroups of anthocyanins, flavanols, and flavanones have been shown to be beneficial in terms of conferring neuroprotection and cognitive benefit. Of note, however, methodological disparities hinder comparison of results between studies. For example, a vast range of flavonoid-containing products utilized in interventions of varying durations, and differences in comprehensiveness of neuropsychological assessments, as well as heterogeneity of cohorts, are all likely to impact findings.

Of the literature reviewed here, acute studies ranged in duration from 60 min to 6 h, whilst the longer-term studies ranged from 28 days to 6 months, with 3 months being the most common timeframe employed. All except two intervention studies included control groups consuming a placebo, with these two studies comparing cognitive function at baseline to end point (16 weeks or 6 h). Whilst studies were included in the current review regardless of placebo being matched with the intervention condition for macro- and micro- nutrient content, this should be a consideration when designing future interventions, to ensure observed effects are due to the flavonoid content and not additional components (e.g., caffeine which has independent effects on alertness, mood, arousal, and concentration). The quantity of flavonoid treatment in the reviewed studies also varied greatly, with some participants consuming 900 mg daily or greater, and others under 100 mg. Interestingly, a study including groups of participants consuming 993 mg or 520 mg daily, for 8 weeks, found both quantities were associated with improvements in measures of attention, visual search and scanning, sequencing and shifting, and psychomotor speed, but only the 993 mg group also demonstrated increased performance in verbal fluency (Mastroiacovo et al., 2015).

**FIGURE 2:**
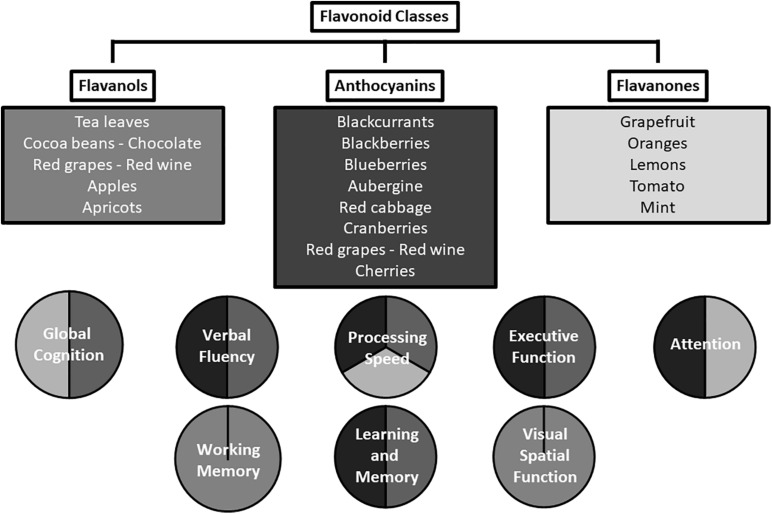
Cognitive domains affected by the flavonoid subclasses of anthocyanins, flavanols, and flavanones.

A range of cognitive domains have been shown to be beneficially affected in the reviewed studies; summarized in Figure 2. Acute supplementation with the three flavonoid subclasses investigated was associated with positive effects in tests measuring psychomotor speed, executive function, and attention. Longer-term supplementation was associated with positive effects in tests of episodic memory, global cognition, verbal fluency, psychomotor speed, and verbal memory, in multiple studies. Although a particular task may have a primary focus such as verbal fluency or episodic memory, a range of processes may support the primary focus, e.g., memory, processing speed, and motor function. Potentially, flavonoids could be exerting greater effects on relatively effortful tasks and less effect on simpler tasks. This notion is consistent with the results of Bondonno et al. (2014), who utilized a computerized cognitive assessment battery which was not heavily loaded with cognitive processing tasks, and yielded no significant associations. Furthermore, Gratton et al. (2020) showed improvements in cognitive function only when cognitive demand is high; significant associations were only observed 2 h following high flavanol consumption in the Double Stroop condition and not in the standard word and color Stroop conditions. A wide range of tasks which fully assess cognitive ability (multiple domains and tasks of varying complexity) needs to be employed by studies attempting to fully characterize the effects of flavonoids on cognition.

The mechanisms by which the flavonoid subclasses of anthocyanins, flavanols, and flavanones modulate cognitive function are yet to be fully established. Available evidence plausibly suggests these flavonoid subclasses can promote beneficial effects via both direct (i.e., through receptor activation, neurotrophin, and signaling pathway modulation) and indirect actions (i.e., enhancement of CBF). Although, it should be noted that the majority of mechanistic studies to date have been conducted in animal models, and more human studies are required to draw definitive conclusions.

Wide inter-individual variability in flavonoid absorption and metabolism has been reported, with a number of factors including age and gender affecting these metabolic processes. Flavonoids initially undergo extensive metabolization by phase I and II metabolism which occur predominantly in the gastrointestinal tract and liver. Hepatic perfusion and morphology reduce as part of the aging process, which has been suggested to reduce phase I and II metabolism of flavonoids. Flavonoid absorption occurs in both the small and large intestines, with a high percentage reaching the colon where there is exposure to colonic microbiota (Williamson and Clifford, 2010). The microbiome plays a key role in catabolizing unabsorbed flavonoids into smaller molecules which may become bioavailable. It is conceivable that heterogeneity in flavonoid absorption and metabolism may diminish beneficial associations between intakes and cognitive outcomes reported in studies. Ideally, urinary flavonoid excretion results would be taken into account in analyses, and to inform personalized health goals.

## Future Research

Overall, additional prospective studies conducted in diverse populations, and adequately powered intervention studies with long durations, are required to thoroughly examine the effect of consumption of the flavonoid subclasses of anthocyanins, flavanols, and flavanones on clinically relevant cognitive outcomes. Moreover, the characterization of appropriate dosage, timeframes for intake, and form of flavonoids, remains to be fully determined. Whilst animal models have shown promising results with interventions of timeframes such as 2 months, this represents a considerably larger percentage of total lifespan for animals than it does for humans, and a longer time-frame could be needed to show enhanced outcomes in human trials. Both acute and chronic effects of these flavonoid subclasses also need to be investigated using neuroimaging techniques in conjunction with cognitive and physiological measures to further elucidate the underlying biological mechanisms. Furthermore, it should be acknowledged that studies in cognitively normal adults are unlikely to demonstrate large improvements in cognitive function, and therefore it is imperative that suitable, sensitive cognitive tests are utilized. There is currently no clear evidence regarding the specific domains of cognition and memory that these flavonoid subclasses impact, with future studies requiring a range of cognitive domains to be investigated in order to determine those most likely to benefit. If emerging evidence continues to suggest significant cognitive benefit, another important consideration is the optimum age for the initiation of supplementation of flavonoid intake. Indeed, the neuropathological hallmarks of AD begin to accumulate 15–20 years before symptoms manifest, implying that the optimal age for supplementing flavonoid consumption could be middle-age or younger.

## Conclusion

As populations continue to focus on developing strategies to promote healthy aging, dietary interventions with flavonoids represents a promising avenue for future research. However, many questions still need to be answered before a definite conclusion can be made regarding the extent to which consumption of anthocyanins, flavanols, and flavanones can protect the aging brain, and intake can be included in public health dietary recommendations.

## Author Contributions

SG and SR-S contributed to the conception and design of the work. SG prepared the first draft of the manuscript. SR-S, MW, CB, and RM revised the manuscript. All authors approved the final version of the manuscript.

## Conflict of Interest

The authors declare that the research was conducted in the absence of any commercial or financial relationships that could be construed as a potential conflict of interest.

## Publisher’s Note

All claims expressed in this article are solely those of the authors and do not necessarily represent those of their affiliated organizations, or those of the publisher, the editors and the reviewers. Any product that may be evaluated in this article, or claim that may be made by its manufacturer, is not guaranteed or endorsed by the publisher.

## Supplementary Material

Supplementary Table 1 Intervention human studies published over the past 6 years investigating flavonoid consumption and cognitive outcomes.
