# Supplementary material for: Intake of Products Containing Anthocyanins, Flavanols, and Flavanones, and Cognitive Function: A Narrative Review
Source: Front Aging Neurosci. 2021 Sep 3;13:640381. doi: 10.3389/fnagi.2021.640381 (PMC8446387; doi:10.3389/fnagi.2021.640381)
Supplement: Supplementary Table 1 — Intervention human studies published over the past 6 years investigating flavonoid consumption and cognitive outcomes. [file Table_1.docx]

**Supplementary Table 1**: Intervention human studies published over the past six years investigating flavonoid consumption and cognitive outcomes

| **Title, Reference** | **Cohort Information** | **Study Design** | **Flavonoids Investigated** | **Outcome Measure(s)** | **Main Finding(s)** |
| --- | --- | --- | --- | --- | --- |
| **Insights into the role of diet and dietary flavonols in cognitive aging: results of a randomised controlled trial [27]** | N = 211  Age: 50 – 75  Cognitively normal  New York, USA | Randomised, controlled, parallel-arm trial  12-week intervention | 260, 510, or 770 mg/day cocoa flavanols or placebo | Objection Recognition, List-learning, List sorting | Intake level-dependent treatment effect compared to placebo on hippocampal-dependent list-learning performance. |
| **Dietary flavanols improve cerebral cortical oxygenation and cognition in healthy adults [28]** | N = 18  Age: 18 – 45  Cognitively normal  Birmingham, UK | Randomised, placebo-controlled, double blind, cross-over design  Acute study | 150 mg epicatechin + 35.5 mg catechin, or placebo < 4 mg of epicatechin and catechin | At 2 hours: Modified Stroop Task | Significantly lower inverse efficiency score in the Double Stroop condition and not in the standard Stroop condition following flavanol intervention. |
| **Flavonoid-rich mixed berries maintain and improve cognitive function over a 6 h period in young healthy adults [22]** | N = 40  Age: 20 – 30  Cognitively normal  Berkshire, UK | Randomised, placebo-controlled single-blind trial  Acute study | 400 ml berry smoothie containing 570 mg flavonoids (strawberry, blueberry, blackberry, raspberry) | At 2, 4 and 6 hours: Executive Function via MANT and TST | Intervention group maintained accuracy in executive function up to and including the 6 hour testing point, and demonstrated quicker response times in the MANT at 2 and 4 hours, and TST at 6 hours testing point. |
| **Effect of Montmorency tart cherry juice on cognitive performance in older adults: a randomized controlled trial [6]** | N = 37  Age: 65 - 80  Cognitively normal  University of Delaware, USA | Randomized controlled trial  12-week intervention | 68 ml Montmorency tart cherry juice, or zero-flavonoid control drink | Cambridge Neuropsychological Test Automated Battery, Paired Associates Learning Test, Rapid Visual Information Processing test, Reaction Time test, Spatial Working Memory test, Digit Span | Intervention group had better performance on the Paired Associates Learning Task which assesses episodic visual memory and new learning, and significantly better movement time scores on the Reaction Time test which measures speed of response and movement to a visual cue, compared to the control group. |
| **Effects of anthocyanin supplementation on serum lipids, glucose, markers of inflammation and cognition in adults with increased risk of dementia – a pilot study [7]** | N = 27  Age: ≥ 50  8 with MCI, 19 with stable non-obstructive coronary artery disease  Norway | Open-label study  16-weeks | Two Medox® capsules per day (daily intake 320 mg anthocyanin from bilberry and blackcurrants)  No control group for the cognitive analysis | Ten Word List Learning and Recall from the CERAD battery, Trail Making Test, Stroop Golden | Improvements in CERAD learning, recall, and recognition (measuring verbal memory) post-intervention.  Improvement in Stroop test word and colour measuring cognitive speed and inhibition post-intervention. |
| **Enhanced neural activation with blueberry supplementation in mild cognitive impairment [25]** | N = 16  Age: 68 - 92  MCI  Cincinatti, Ohio, USA | Randomised, double-blind placebo-controlled trial  16-weeks | Blueberry anthocyanins 269 mg or placebo | Working memory *n*-back task | No significantly improved working memory task performance |
| **Polyphenols from grape and blueberry improve episodic memory in healthy elderly with lower level of memory performance: A bicentric double-blind, randomised, placebo-controlled clinical study [39]** | N = 215  Age: 60 - 70  Cognitively normal  France and Canada | Bicentric double-blind, randomised, placebo-controlled intervention  6 months | Flavanol-rich extract from grape and blueberry (258 mg flavonoids daily) or placebo | CANTAB PAL, VRM - free recall and delayed recognition, working memory | Improvements in VRM-free recall with no effect on PAL.  With the cohort stratified into quartiles using baseline PAL, a subgroup with advanced cognitive decline had better VRM-delayed recognition. |
| **Dietary blueberry improves cognition among older adults in a randomized, double- blind, placebo-controlled trial [21]** | N = 37  Age: 60 - 75  Cognitively normal  Boston, Massachusetts, USA | Randomized, double-blind, placebo controlled trial  90-day intervention | 24 g freeze-dried blueberries (contains 19.2 mg/g anthocyanins) or a placebo | At 45 and 90 days: Task Switching Test, Trail Making Test, Digit Span, California Verbal Learning Test (2^nd^ Edition), Virtual Morris Water Maze, Attention Network Task | Participants in the control group made more, and participants in the blueberry group made fewer, repetition errors on California Verbal Learning Test free recall (measure of verbal memory) at day 90 than at baseline.  Intervention group showed reduced switch stimuli errors (measure of executive function) on task switching test across study visits relative to controls. |
| **Acute effects of flavonoid rich blueberry on cognitive and vascular function in healthy older adults [23]** | N = 18  Age: 62 - 73  Cognitively normal  Berkshire, UK | Cross over randomised controlled trial  Acute study | Flavonoid-rich beverage drink (579 mg of antho- and pro-cyanidins), or sugar-matched control | At 2 and 5 hours:  Go-No-Go, Stroop, Digit Switch, Continuous Performance Task, Digit Symbol Substitution Test, Random Word Generation, Three Word Sets Task, N-back, Letter Memory, Location Task, Immediate and Delayed Recall and Recognition | Decline in performance in the control group from the 2-hour to the 5-hour assessment.  No significant difference in cognitive performance at 2- compared to 5-hour assessment post- consumption of the blueberry beverage. |
| **Cognitive response to fish oil, blueberry, and combined supplementation in older adults with subjective cognitive impairment [24]** | N = 65  Age: 62 - 80  Mild, self-perceived cognitive decline  Cincinatti, Ohio, USA | Randomized, double-blind, parallel groups, placebo controlled trial  24-week intervention | Daily fish oil; daily blueberry (providing 269 mg anthocyanin per day); both fish oil and blueberry; or control | At 24 and 48 weeks: Trail Making Test, Controlled Oral Word Production, Hopkins Verbal Learning Test | Improved discrimination in recognition memory on the Hopkins Verbal Learning Test for the blueberry-treated group, at the end of the intervention, however this was not maintained at week 48. |
| **A randomized, double- blinded, placebo controlled study to compare the safety and efficacy of low dose enhanced wild blueberry powder and wild blueberry extract (ThinkBlue^TM^) in maintenance of episodic and working memory in older adults [20]** | N = 112  Age: 65 - 80  Cognitively normal  Berkshire, UK | Randomized, double-blind, placebo controlled trial  6-month intervention | Placebo or one of three interventions:  whole wild blueberry powder (WBP500; 1.35 mg anthocyanin), whole wild blueberry powder (WBP1000; 2.7 mg anthocyanin), or purified extract (WBE111; 7 mg anthocyanin) | At 3 and 6 months:  Rey Auditory Verbal Learning Test, Object Recognition Task, Corsi Blocks Task, Sternberg Memory Scanning Test, Serial Subtractions Task, Modified Attention Network Task, Stroop | 3-month testing point: delayed word recognition on the Rey Auditory Verbal Learning Test and performance on the Corsi Blocks Task (measurement of short-term spatial episodic memory) were significantly better following the WBE111 treatment compared to control.  There were no significant differences at the 6-month cognitive assessments, or with consumption of the WBP500 and WBP1000 interventions. |
| **Enhanced task-related brain activation and resting perfusion in healthy older adults after chronic blueberry supplementation [26]** | N = 26  Age: 69.0 ± 0.9 (placebo) 67.5 ± 0.9 (intervention)  Cognitively normal  Exeter, UK | Double-blind randomized controlled trial  12 weeks | 30 mL blueberry concentrate (389 mg anthocyanin) or sugar matched control | Cogstate Ltd battery assessing psychomotor function, visual processing, executive function, verbal and spatial memory, and working memory. Numerical Stroop test assessing attention and memory | Change in performance on cognitive tests were not significantly different between groups. The percentage change in performance on the *2*-back test showed weak evidence for improvement in the blueberry versus placebo group. |
| **Brachial and cerebrovascular functions are enhanced in postmenopausal women after ingestion of chocolate with a high concentration of cocoa [36]** | N = 12  Age: 57.3 ± 5.3  Cognitively normal  Perth, Australia | Counter-balanced within-subject design  Acute study | White chocolate (370 mg/kg flavonoids), milk chocolate (980 mg/kg flavonoids), dark chocolate (3600 mg/kg flavonoids) | At 1 hour: CogState computer-based battery including detection task, identification task, “1 back” and “2 back” tasks, the international shopping list learning and recall, continuous paired association learning | No significantly improved performance. |
| **Cognitive and mood improvements following acute supplementation with purple grape juice in healthy young adults [40]** | N = 20  Age: 18 - 35  Cognitively normal  Newcastle upon Tyne, UK | Randomised, placebo-controlled, double blind, counterbalanced-crossover trial | 230 mL purple grape juice (138 mg anthocyanin) or sugar matched control | At 20 minutes: composite scores for memory accuracy, memory reaction time, attention accuracy, attention reaction time | Significantly improved reaction time on the composite attention measure in the grape juice group. |
| **Consumption of anthocyanin-rich cherry juice for 12 weeks improves memory and cognition in older adults with mild-to-moderate dementia [16]** | N = 49  Age: 70+  Mild-to-moderate dementia  Illawarra region of New South Wales, Australia | Randomized controlled trial  12-weeks | Cherry juice (138 mg anthocyanin), or apple juice (0.04 mg anthocyanin) | At 6 and 12 weeks: Rey Auditory Verbal Learning Test, Self-ordered Pointing Task, Trail Making Test, Boston Naming Test, Digit Span Backwards Task, Category and Letter Verbal Fluency | Significant improvements in the verbal fluency task, and Rey Auditory Verbal Learning Test total, delayed recall, and 20-minute delayed recall were observed in the cherry juice consumption group. |
| **The effects of flavanone-rich citrus juice on cognitive function and cerebral blood flow: an acute, randomised, placebo-controlled cross-over trial in healthy, young adults [43]** | N = 24  Age: 18 - 30  Berkshire, UK | Single blind, randomised, cross over design  Acute study | High flavanone drink (70.5 mg) and zero-flavanone drink | At 2 hours: Freiburg Vision Test, Word Recall Immediate and Delayed, Logical Memory Immediate and Delayed, Sequence Learning Task, Digit Symbol Substitution Task, Stroop test, Letter Memory Test, Go-No-Go computerised task, and Spatial Delayed Recall | Consumption of high flavanone drink resulted in improvement in Digit Symbol Substitution Task score at 2 hours post-consumption, relative to baseline and the control drink. |
| **Flavanol-rich chocolate acutely improves arterial function and working memory performance counteracting the effects of sleep deprivation in healthy individuals [31]** | N = 32  Mean age: 25.31  L’Aquila, Central Italy | Two baseline sessions after one night of undisturbed sleep.  Two experimental sessions after one night of total sleep deprivation | Flavanol-rich chocolate treatment (520 mg total flavanols)  Flavanol-poor chocolate treatment (88.5 mg total flavanols) | At 2 hours: Psychomotor vigilance task  Working memory task - 2-back task | In females, the accuracy on 2-back task in the sleep deprivation condition was higher after the flavanol-rich treatment in comparison to the flavanol-poor treatment. |
| **High-flavonoid intake induces cognitive improvement linked to changes in serum brain-derived neurotrophic factor: Two randomised, controlled trials [30]** | Trial 1:  N = 154  Age: 26 - 70  Consuming less than 4.4 portions of fruit and vegetable servings per day  UK  Trial 2:  N = 40  Age: 62 - 75  Cognitively normal  Berkshire, UK | Trial 1:  Randomized, controlled, dose-dependent, parallel designed trial  18-week intervention  Trial 2:  Randomised, controlled, double-masked, crossover  28-day intervention | Trial 1:  High flavonoid fruit and vegetables, low flavonoid fruit and vegetables, and control (habitual diet) Incrementally increasing 2, 4, and 6 portions of fruit and vegetable intake every 3 weeks  Trial 2:  High flavanol cocoa drink (494 mg) and low flavanol cocoa drink (23 mg) | At 6, 12, and 18 weeks: Global cognition | Trial 1:  High flavonoid intervention significantly improved global cognition at 12- and 18-week assessments relative to both the low flavonoid and control diets.  Trial 2:  High flavanol cocoa drink led to significantly increased global cognitive performance compared to low flavanol cocoa drink consumption. |
| **Anthocyanin-rich cherry juice does not improve acute cognitive performance on RAVLT [18]** | N = 13  6 aged 18 - 35  2 cognitively normal aged 55+  5 with dementia aged 55+ | Pilot cross-over study | Cherry juice (anthocyanin 55 mg), single 300 ml serving at 0 hour, or 3 x 100 ml servings at 0, 1, and 2 hours | At 6 hours: Rey Auditory Verbal Learning Test, Task switching, pattern and letter comparison | Task switching test was statistically different after consumption of the whole 300 ml serving in cognitively healthy older adults. |
| **Flavonoid-rich orange juice is associated with acute improvement in cognitive function in healthy middle-aged males [42]** | N = 22  Age: 30 - 65  Males  Berkshire, UK | Randomized, double blind, counterbalanced order  Acute study | Flavonoid rich (220.46 mg hesperidin, 34.54 mg narirutin, and other flavonoids 17.14 mg) and zero-flavonoid drink | At 2 and 6 hours: Immediate and Delayed Word Recall, Simple and Complex Finger Tapping, Digit Symbol Substitution Task, Continuous Performance Task, Serial Sevens, and Contrast Sensitivity | Performance on Simple Finger Tapping and Continuous Performance Task accuracy (tests of executive function and psychomotor speed) were significantly better following flavonoid-rich drink, compared to placebo, at the 6-hour post-consumption assessment. |
| **Acute cocoa flavanol improves cerebral oxygenation without enhancing executive function at rest or after exercise [35]** | N = 20  Age 30 ± 3  Males  Belgium | Randomised, double-blind, crossover design  Acute study | Flavanol rich (904 mg), and control drink (15 mg flavanol) after 100 minutes completed 30-minute time trial | Stroop task | No significantly improved performance. |
| **Acute supplementation with blackcurrant extracts modulates cognitive functioning and inhibits monoamine oxidase-B in healthy young adults [19]** | N = 36  Age: 18 - 35  Auckland, New Zealand | Randomised, double-blind, placebo-controlled, crossover study  Acute study | Anthocyanin-enriched blackcurrant extract (7.79 mg per kg of body weight), cold-pressed blackcurrant fruit juice (8.05 mg per kg of body weight), and zero-anthocyanin control | At 70 minutes: Seven repetitions of the Digit Vigilance task, Stroop task, and Rapid Visual Information Task,  Logical Reasoning | Increase in Digit Vigilance speed of response after juice treatment at repetitions 1 and 7.  Attenuation in the reduction of Rapid Visual Information Task accuracy following the extract treatment compared with control. |
| **Chronic consumption of flavanone rich orange juice is associated with cognitive benefits: an 8-wk, randomized, double blind, placebo-controlled trial in healthy older adults [41]** | N = 37  Age: 60 - 81  Cognitively normal  Berkshire, UK | Double blind, randomized, crossover design  8-week intervention | High flavanone orange juice (305 mg), or low flavanone orange-flavoured cordial drink (37 mg) | Executive Function: Go-No-Go computerised task, Serial Sevens, Digit Symbol Substitution Test, Letter Fluency, and Letter Memory  Episodic Memory: CERAD immediate verbal recall, delayed verbal recall, Verbal Paired Associates immediate and delayed, and Spatial Working Memory | Global performance (all cognitive tests combined into one score) was significantly better after high flavanone juice than after low flavanone drink.  Performance was significantly better when high flavanone juice was consumed during first arm than when low flavanone drink was consumed during first arm (may have carryover effects).  Rather than improve cognitive function, the flavanone-rich juice attenuated general decline in performance observed after 8 weeks of consumption of low flavanone drink. |
| **Cocoa flavanol consumption improves cognitive function, blood pressure control, and metabolic profile in elderly subjects: the Cocoa, Cognition, and Aging (CoCoA) Study – a randomized controlled trial [33]** | N = 90  Age: 61 - 79  Without clinical evidence of cognitive dysfunction  L’Aquila district, Central Italy | Randomized controlled trial double-blind  8-week intervention | High flavanol drink (993 mg), intermediate flavanol drink (520 mg), and low flavanol drink (48 mg) | MMSE  Trail Making Test  Verbal Fluency Test | Mean change in completion time of Trail Making Test after consumption of the high flavanol and intermediate flavanol drinks significantly differed from that after consumption of the low flavanol drink.  Verbal Fluency Test scores significantly improved among all treatment groups, but magnitude of improvement was higher in high flavanol group compared to intermediate and low drink consumption. |
| **The acute and sub-chronic effects of cocoa flavanols on mood, cognitive and cardiovascular health in young healthy adults: a randomized, controlled trial [34]** | N = 40  Age: 18 - 40  Cognitively normal  Melbourne, Australia | Randomized controlled trial, parallel design  Acute and sub-chronic study | Cocoa tablet (250 mg cocoa flavanols) or placebo tablet | At 2 - 3.5 hours and 4 weeks: Swinburne University Computerized Cognitive Assessment Battery  Three repeated 10-minute cycles of the Cognitive Demand Battery encompassing 2 Serial Subtraction tasks, a Rapid Visual Information Processing Task, and a Mental Fatigue Scale | Acute Assessment: In the first repetition of the Cognitive Demand Battery Serial Sevens task, intervention group provided significantly more correct answers than those on placebo. |
| **The acute effect of flavonoid rich apples and nitrate rich spinach on cognitive performance and mood in healthy men and women [38]** | N = 30  Mean age: 47.3 ± 13.6  Perth, Australia | Randomised controlled cross-over (latin square) design  Acute study | Four interventions:  Control (low flavonoid + low nitrate), apple (high flavonoid + low nitrate), spinach (low flavonoid + high nitrate), apple and spinach (high flavonoid + high nitrate)  Apple contains (-)-epicatechin and flavonol quercetin | At 150 minutes: Cognitive Drug Research computerized cognitive assessment battery | No improvement or deterioration in cognitive function following interventions. |
| **Enhancing dentate gyrus function with dietary flavanols improves cognition in older adults [32]** | N = 37  Age: 50 - 69  Cognitively normal  Columbia University Medical Center/New York Presbyterian Hospital campus | Randomized controlled trial  3-month intervention | High dietary flavanol (900 mg) + exercise,  high dietary flavanol (900 mg) + control,  low dietary flavanol (45 mg) + exercise,  low dietary flavanol (45 mg) + control | ModBent task  Modified Rey Auditory Learning Test | High flavanol intervention had a significant effect on Modbent reaction time performance independent of exercise but had no effect on delayed retention. |

Abbreviations: CANTAB PAL, Cambridge Neuropsychological Test Automated Battery Paired Associates Learning; CERAD, Consortium to Establish a Registry for Alzheimer's Disease; MANT, Modified Attention Network Task; MCI, Mild Cognitive Impairment; MMSE, Mini-Mental State Examination; RAVLT, Rey Auditory Verbal Learning Test; TST, Task Switch Task; VRM, verbal episodic and recognition memory.
